# Tracking Mangrove Oil Bioremediation Approaches and Bacterial Diversity at Different Depths in an *in situ* Mesocosms System

**DOI:** 10.3389/fmicb.2019.02107

**Published:** 2019-09-13

**Authors:** Laís Feitosa Machado, Deborah Catharine de Assis Leite, Caio Tavora Coelho da Costa Rachid, Jorge Eduardo Paes, Edir Ferreira Martins, Raquel Silva Peixoto, Alexandre Soares Rosado

**Affiliations:** ^1^Institute of Microbiology Paulo de Góes, Federal University of Rio de Janeiro, Rio de Janeiro, Brazil; ^2^Research Center Leopoldo Américo Miguez de Mello, Rio de Janeiro, Brazil; ^3^IMAM-AquaRio – Rio de Janeiro Aquarium Research Center, Rio de Janeiro, Brazil

**Keywords:** bioremediation, mangrove, mesocosm, oil-degrading genes, bacterial diversity

## Abstract

In this study, oil spills were simulated in field-based mangrove mesocosms to compare the efficiency of bioremediation strategies and to characterize the presence of the *alk*B, *ndo*, *ass*A, and *bss*A genes and the ecological structures of microbial communities in mangrove sediments at two different depths, (D1) 1–10 cm and (D2) 25–35 cm. The results indicated that the hydrocarbon degradation efficiency was higher in superficial sediment layers, although no differences in the hydrocarbon degradation rates or in the abundances of the *alk*B and *ndo* genes were detected among the tested bioremediation strategies at this depth. Samples from the deeper layer exhibited higher abundances of the analyzed genes, except for *ass*A and *bss*A, which were not detected in our samples. For all of the treatments and depths, the most abundant phyla were Proteobacteria, Firmicutes and Bacteroidetes, with Gammaproteobacteria, Flavobacteriales and Clostridiales being the most common classes. The indicator species analysis (ISA) results showed strong distinctions among microbial *taxa* in response to different treatments and in the two collection depths. Our results indicated a high efficiency of the monitored natural attenuation (MNA) for oil consumption in the tested mangrove sediments, revealing the potential of this strategy for environmental decontamination and suggesting that environmental and ecological factors may select for specific bacterial populations in distinct niches.

## Introduction

Mangrove forests are known to store a large amount of organic carbon and therefore have the potential to substantially contribute to carbon emissions when exposed to high rates of pollution, degradation and/or deforestation (Hamilton and Friess, 2018). Among the numerous sources of pollution that can affect mangroves, oil spills are one of the most important concerns because they can occur at any stage of the oil production chain and result in extensive environmental and health damage (Santos et al., 2011). Although oil spills can directly affect almost any ecosystem, coastal areas such as mangroves are among the most threatened environments (Duke, 2016). Because of their ecotonal location and structural peculiarities, mangroves typically retain oil from spillages, concentrating hydrocarbons in their surroundings and compromising their physical, chemical and biological systems for long periods of time (Lewis et al., 2011; Duke, 2016).

Several physical and chemical remediation strategies have been used for oil decontamination, but considering their high financial and environmental costs, bioremediation approaches have become increasingly useful for this purpose (Xu and Lu, 2010). Bioremediation can be applied both *in situ* and *ex situ* using different strategies, such as bioattenuation, bioaugmentation and biostimulation. Bioattenuation, also known as monitored natural attenuation (MNA), is an *in situ* technique that consists of the contaminant degradation by natural physical, chemical and biological processes without any human intervention, through volatilization and degradation of the toxic compounds by the autochthonous microbiota. In general, it is a slow process and requires site monitoring over a long period (Khan et al., 2004; Yu et al., 2005). Bioaugmentation consists of the injection of microorganisms grown in the laboratory, autochthonous from the contaminated site, with known ability to degrade the target contaminant. It can be used *in situ* and *ex situ*, being particularly important for *in situ* treatments, when the autochthonous microbiota is not presented in sufficient abundance to degrade the contaminant efficiently (Desay et al., 2010). Biostimulation, in turn, is the process by which an intentional stimulation of autochthonous microbial communities takes place through the provision of nutrients and electron acceptors/donors, pH and temperature adjustment and aeration of the substrate. This technique can be performed *in situ* and *ex situ*, but depends on the existence of an autochthonous microbiota capable of degrading the target contaminant (Khan et al., 2004; Desay et al., 2010).

Bioremediation techniques have been successfully applied worldwide in environmental oil mitigation, such as in the oil spills in Prince William Sound, Alaska, in 1989 (Bragg et al., 1994) and in the Mexican Gulf in 2010 (Atlas, 2011), and they are promising strategies for cleaning up oil-contaminated mangrove sediments (Santos et al., 2011; Duke, 2016). However, in contrast to most soils, mangrove sediments are predominantly anaerobic, which should be considered when attempting to develop a bioremediation strategy (Ghizelini et al., 2012).

Some studies have examined the microbial degradation of pollutants to address questions related to details of the bioremediation process, such as transcriptional information and kinetic behavior associated with enzymes involved in pollutant degradation (Peixoto et al., 2011a; Thatoi et al., 2013). The results of these studies have provided significant insights for improving bioremediation processes. However, most studies analyzing the effect of hydrocarbon pollution on subtidal sediments have disregarded the differential distribution of bacteria throughout the depth profile, and sediment samples have generally been analyzed in bulk or sampling has been primarily focused in the upper layer (0–5 cm), where metabolism is primarily aerobic. Other assays, such as the characterization of syntrophic pollutant-degrading microorganisms and the detection of genes that are potentially involved in the bioremediation processes of microbial communities, are still of great importance. For oil bioremediation purposes, some bacterial groups have already been recognized for their ability to metabolize oil hydrocarbons, such as members of the genera *Acinetobacter*, *Alcaligenes*, *Paenibacillus*, and *Pseudomonas* (do Carmo et al., 2011; Kostka et al., 2011; Santos et al., 2015). Some genes associated with the outset of hydrocarbon metabolism have also been identified, such as *alk*B (encoding alkane monooxygenase) and *ndo* (encoding naphthalene dioxygenase), which are active under aerobic conditions to degrade alkanes and polycyclic aromatic hydrocarbons (PAHs), respectively (Beilen et al., 1994; Gomes et al., 2007), as well as *ass*A (encoding alkylsuccinate synthase A) and *bss*A (encoding benzylsuccinate synthase A), which are active under anaerobic conditions to degrade alkanes and PAHs, respectively (Andrade et al., 2012; Netzer et al., 2013).

Mangroves are permanently vulnerable to potential oil contamination. Previous studies have shown that the microbiome of these ecosystems can be heterogeneously distributed throughout the mangrove (Peixoto et al., 2011b) and is affected by the concentration of oil (Santos et al., 2010, 2011; Fasanella et al., 2012), presenting great metabolic versatility with enzymatic potential for the degradation of hydrocarbons (Santos et al., 2010). The use of native microbial species or consortia for mangrove bioremediation can represent a promising and efficient approach for the recovery of these ecosystems. However, the deposition and adsorption of oil in the subsurface layers of mangrove sediments is a problem that current bioremediation approaches do little to help solve. Therefore, the utilization of a microbial consortium containing facultative anaerobic bacteria has been suggested as a potential strategy to overcome this limitation (Andrade et al., 2012).

In our study, we selected and assembled an oil degrading bacterial consortium containing local mangrove native aerobic and facultative anaerobic bacteria and developed an innovative *in situ* mesocosm system in a mangrove located in the state of Rio de Janeiro, Brazil. This innovative mesocosm system was simultaneously safe against environmental contamination and dynamic to generate a realistic system that included tidal influence. After a local and controlled oil spill simulation in the mesocosms, we evaluated: (i) the presence of different hydrocarbon degradation genes during the oil contamination and bioremediation processes; (ii) oil degradation in response to different bioremediation processes; (iii) differences in bacterial diversity patterns along the sediment depth at this site.

## Materials and Methods

### Experimental Design

#### Mesocosm Construction

**FIGURE 1:**
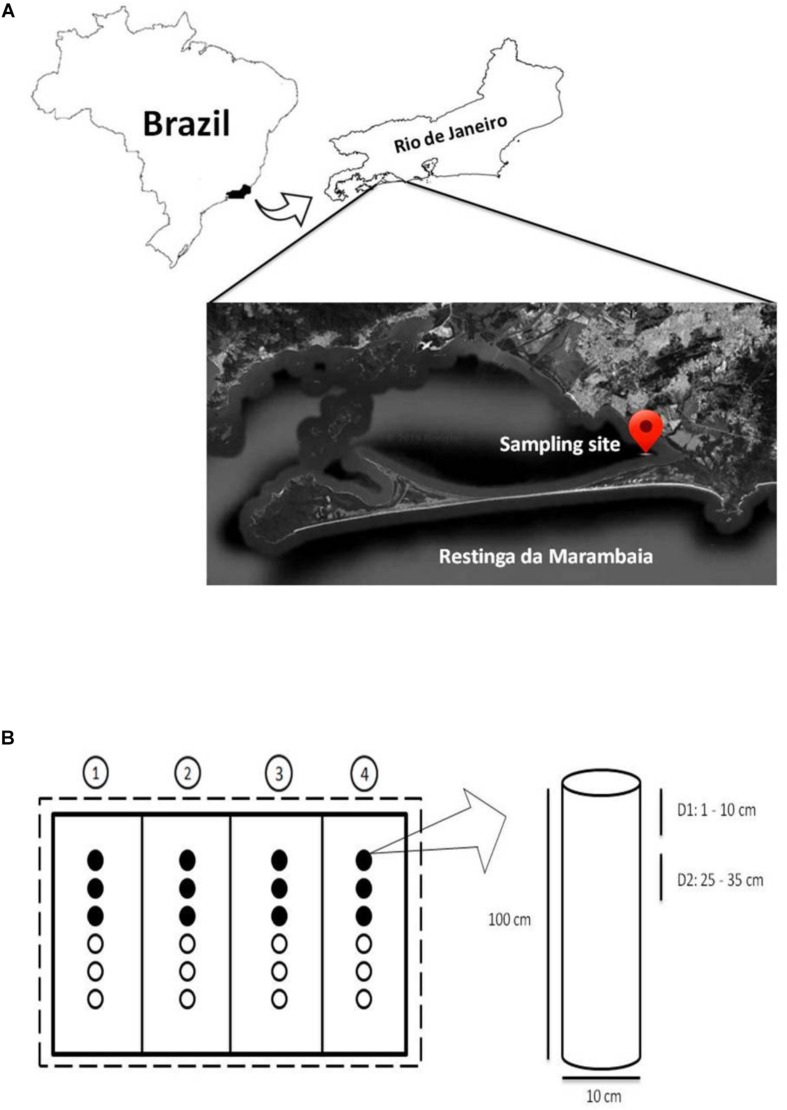
**(A)** Sampling location (modified from Google Earth – view from 72 km altitude). **(B)** Experimental design outline. Dashed line: contention barrier; solid lines: absorption barriers; circles: mesocosms. The numbers in circles represent the bioremediation treatments applied in each set of mesocosms: 1, Monitored Natural Bioattenuation; 2, Bioaugmentation; 3, Biostimulation; 4, Bioaugmentation + Biostimulation. Black and white circles represent samples collected after 45 and 90 days of bioremediation treatments application, respectively. The cylinder represents a mesocosm with the two collection depths: depth 1 (D1) 1–10 cm and depth 2 (D2) 25–35 cm. Not drawn to scale.

A mesocosm experiment was developed (Figure 1 and Supplementary Figure S1) at the mangrove of Restinga da Marambaia, Rio de Janeiro, Brazil (23°03′18.7″S/43°34′19.7′′W; Permitted by SisGen AC72B83, according to the Brazilian legislation on access to biodiversity (Law 13,123/15 and Decree 8.772/16) to test bioremediation techniques against the MF (marine fuel) 380 oil (composed of mixtures of medium and heavy fractions of fuel oil, with paraffinic and aromatic hydrocarbons of different molecular weights). Aluminum cylindrical tubes (100 cm in length and 10 cm in diameter) were used as mesocosms (Figures 1A,B and Supplementary Figure S1) and vertically inserted into mangrove sediments. Twenty-four tubes were used in this study that were divided in four groups, one for each treatment, and arranged in a linear configuration, with six tubes per group. Each group was surrounded by oil absorption barriers, and the total experimental area was physically separated from the rest of the mangrove by oil contention barriers (Supplementary Figure S1). To ensure the efficiency of the barriers despite tidal hydrodynamics, these structures were affixed to iron brackets with a length of 4 m (calculated based on the maximum tidal range) using metallic rings to provide them vertical movement. This movement allowed them to follow water levels, improving the protection efficiency in the experimental area, improving protection efficiency in the experimental area.

#### Bioremediation Strategies

The oil used in this study was the marine fuel MF-380, which has the following characteristics: density (0.9817), 12°API, viscosity of 5313 cP at 20°C, boiling point range from 174 to 750°C (26%, w/w residue), BTEX content of 1.56 mg.g^–1^ and 50 mg.g^–1^ PAHs. Four different bioremediation techniques were tested to mitigate the MF-380 oil spill simulation: Bioattenuation (BT), Bioaugmentation (BA), Biostimulation (BS) and Bioaugmentation + Biostimulation (BB). For the BA and BB strategies, a bacterial consortium was selected and assembled. This consortium was composed of native hydrocarbon-utilizing bacteria isolated under anaerobic and aerobic conditions. The aerobic bacteria used in this study were previously obtained by do Carmo et al. (2011) in the exactly same experimental area and have been classified at the molecular level as *Acinetobacter* sp., *Alcaligenes* sp., *Azospirillum* sp., *Paenibacillus* sp., *Pseudomonas* sp., *Pseudomonas pseudoalcaligenes*, and *Pseudomonas stutzeri*. The sequences from the aerobic bacteria are available at the NCBI Sequence Read Archive under the following accession numbers: HQ020607, HQ020582, HQ020562, HQ020564, HQ020609, HQ020568, and HQ020576, respectively. The anaerobic strains used in this study were isolated from mangrove sediments at the same sampling site (23°03′18.7′′S/43°34′19.7′′W). Sediment samples were collected at a depth of 15–75 cm in the syzygy tidal zone with a cylindrical sampler (7.5 cm in diameter and 75 cm in length). Subsequently, the these samples were blended, and 12.5 g was homogenized in 125 mL of a sterile reducing buffer in an anaerobic culture system. Serial dilutions (10^–1^ to 10^–4^) were generated and 100 μL aliquots were used to inoculate Bushnell-Hass broth (MgSO_4_, CaCl_2_, KH_2_PO_4_, K_2_HPO_4_, (NH_4_)(NO_3_), and FeCl_3_) that were cultured in triplicate under the following conditions: pH 6, 7, and 8, and salinity of 0.5 and 2%. The bacterial cultures were incubated at 27°C. All media were supplemented with MF 380 oil as the sole carbon source. After 30 days, these cultures were inoculated into Petri dishes containing marine agar medium (Marine Agar Zobell 2216, Himedia Laboratories, Mumbai, India) and incubated at 27°C. The cultured strains were selected and identified using molecular techniques before becoming part of the consortia. For this step, genomic DNA was extracted using a Wizard^®^ Genomic DNA Purification Kit (Promega, Madison, United States) following the manufacturer’s instructions. The resulting DNA was used for PCR targeting the 16S rRNA gene with the primers 27F (AGA GTT TGA TCA TGC CTC AG), and 1492R (GTT TAC CTT GTT ACG ACT T). The PCR products were purified, sequenced, analyzed using the Ribosomal Database Project (RDP) (Cole et al., 2009) and compared with sequences deposited at the National Center for Biotechnology Information (NCBI)^1^ using the BLAST tool (Basic Local Alignment Search Tool) for taxonomic classification. Antagonistic tests were performed between all strains according to Giambiagi-Marval et al. (1990). These tests are important to identify antagonistic strains that should not be used simultaneously in a given consortium. Strains that did not present antagonism against each other were selected to be part of the consortium. The number of cells of each individual strain was estimated by counting the number of colony forming units (CFUs) over time using serial dilutions (10^3^–10^7^) in triplicate. For each dilution, 200 μL was spread onto Petri dishes containing marine Agar, and the resulting colonies were counted using a colony counter. Subsequently, the aerobic and anaerobic strains were inoculated into 300 mL of Luria–Bertani broth and Baar broth medium (Na_3_C_6_H_5_O_7_, MgSO_4_, CaSO_4_, NH_4_Cl, K_2_HPO_4_, sodium lactate, yeast extract and Fe(NH_4_)_2_(SO_4_)_2_), respectively, and were incubated for 48 h. The aerobic strains were incubated at 150 rpm, whereas the anaerobic strains were incubated without rotation to avoid aeration of the medium. The bacterial cultures were centrifuged at 5000 rpm for 30 min, and the resulting cell pellets were resuspended in 60 mL of NaCl 0.9% to obtain a density of 10^7^ viable cells mL^–1^. Equal volumes of each bacterial isolate suspension were mixed, yielding a consortium containing 10^7^ cells mL^–1^ of each selected strain. To evaluate the bioremediation strategies, each tube of the mesocosm was vertically contaminated with 90 mL of MF 380 oil (1.4%) using a syringe manufactured specifically for this purpose (Supplementary Figure S2A). The syringe had a piston with a length of 1.20 m and a cylinder of 1.10 m, with a capacity of 90 mL. The widest and narrowest points of the syringe were 10 and 4 mm across, respectively. To ensure that oil would be equally distributed in all sediment layers, we developed a isosceles triangle tool that had the largest intersection lines measuring 1.35 m, a base of 0.65 m, and a height of 1.30 m. To apply the oil, the triangle tool was placed above each mesocosm, and the syringe containing the oil was inserted into the metallic tubes until the piston fit perfectly to the upper vertex of the triangle tool. With the piston in the upper triangle vertex, the syringe was slowly removed from the mesocosm (Supplementary Figure S2B).

Four months after oil contamination, the bioremediation strategies were applied. In each tube, 90 mL of each treatment was vertically applied using the same strategy used for oil contamination. The treatments consisted of the following strategies: (1) Monitored Natural Bioatenuation: the application of no treatment to allow the catabolic activity of local microorganisms, with no other stimulation other than those from the natural environment and monitoring; (2) Bioaugmentation: the application of 45 mL of the bacterial oil-degrading consortium (10^7^ cell/mL) under aerobic and anaerobic conditions plus 45 mL of 0.9% sterile saline; (3) Biostimulation: the application of 45 mL of a nitrogen, phosphorous and potassium (NPK) solution in a 18:2:10 (w/w) proportion (Ramsay et al., 2010) plus 45 mL of 0.9% sterile saline; (4) Bioaugmentation + Biostimulation: 45 mL of the bacterial oil-degrading consortium under aerobic and anaerobic conditions (10^7^ cell/mL) plus 45 mL of NPK in a 18:2:10 (w/w) proportion. After 45 and 90 days of the application of the different treatments, three tubes were collected from each treatment and processed. The sediment columns were sectioned in two depth fractions, 1–10 cm (depth 1; D1) and 25–35 cm (depth 2; D2), and each fraction was manually homogenized and used for further analyses.

### Physical and Chemical Characterization of Sediment

Sediment samples were characterized for their granulometry and carbon and nitrogen contents. Sediment granulometry was determined using the aerometer method after chemical dispersion (EMBRAPA, 2011). Carbon content was evaluated via the oxidation of wet organic matter with potassium dichromate in sulfuric medium using the heat released from the sulfuric acid as a heat source. The excess dichromate after oxidation was titrated with a standard solution of ammoniacal ferrous sulfate (EMBRAPA, 2011). To determine the total nitrogen content, N was converted to (NH_4_)_2_SO_4_ by oxidation with a mixture of CuSO_4_, H_2_SO_4_ and Na_2_SO_4_ or K_2_SO_4_. Subsequently, the (NH_4_)_2_SO_4_ was placed in an alkaline medium in a diffusion chamber, where it released ammonia that complexed with a boric acid solution containing a mixed indicator and then was measured by acidimetry (with H_2_SO_4_ or HCl) (EMBRAPA, 2011).

### Evaluation of Hydrocarbon Degradation

The efficiency of the bioremediation strategies was tested by evaluating hydrocarbon degradation. The saturated hydrocarbon content (*n*-alkanes) was evaluated by gas chromatography and flame ionization detection (GC/FID) following the United States EPA Standard Methods for the Examination of Water and Wastewater (846-8015). The detection of polyaromatic hydrocarbons was performed according to the United States EPA Standard Method 846-8270, which describes the use of gas chromatography for semi-volatile organic compounds and mass spectrometry (GC/MS). Hydrocarbon analysis was performed at Oceanus Hidroquímica facility^2^.

### DNA Extraction

Total DNA was extracted from sediment samples (0.5 g) using a Power Soil^TM^ DNA Isolation kit (MoBio^TM^, United States) following the manufacturer’s instructions. The DNA was quantified using a fluorometer (QuBit^®^ Fluorometric Quantification, Life Technologies^TM^, United States) and visualized on a 1% (w/v) agarose gel with SYBR-Safe DNA gel stain (Invitrogen, Life Technologies^TM^, United States) to assess its integrity prior to PCR and qPCR analysis.

### qPCR for Hydrocarbon-Degrading Genes

qPCR was performed to assay the hydrocarbon-degrading *alk*B, *ndo*, *ass*A, *bss*A, and 16S rRNA genes in each sample. qPCR was performed in a 20-μL reaction with 2 × Master Mix (Fermentas), 5 μM of each primer and 2–5 ng of template. In the *ndo* reaction, 1% of dimethyl sulfoxide was added. qPCR was performed under previously described conditions for each set of primers (Supplementary Table S1). At the end of each reaction, we obtained a denaturation curve by heating the products at 95°C for 15 s, followed by a cooling step of 60°C for 1 min and subsequent heating at 95°C for 15 s. All amplifications were performed in duplicate using a 7500 Real-Time PCR System (Applied Biosystems, Germany).

### Bacterial Community Structure Analysis

Bacterial communities were assessed by high-throughput sequencing targeting the V4 variable region of the 16S rRNA gene using the primers 515F (5′-GTG CCA GCM GCC GCG GTA A 3′) and 806R (5′ GGA CTA CHV GGG TWT CTA AT 3′) (Caporaso et al., 2012) with specific adapters. The following thermocycling conditions were used: 94°C for 3 min, followed by 30 cycles of 94°C for 30 s, 53°C for 40 s, and 72°C for 1 min, with a final elongation step at 72°C for 5 min. Equimolar amounts of the PCR products from the 48 samples were combined and sequenced via Ion Torrent Personal Genome Machine (PGM) technology following the manufacturer’s guidelines at the MrDNA facility^3^. Raw sequences were processed using Mothur v.1.36.1 (Schloss et al., 2009). To reduce sequencing error, all sequences that failed to comply with any of the following criteria were excluded: average quality lower than 20, length less than 200 bases, presence of ambiguities, more than 1 nucleotide mismatch to the primer and/or barcodes, or homopolymers longer than 8 nucleotides. The remaining high quality sequences were aligned using the Silva reference database (Quast et al., 2013), and chimeras that were detected using Uchime (Edgar et al., 2011) were eliminated. The resulting alignment file was used as input to construct a distance matrix and to cluster the sequences into operational taxonomic units (OTUs), with a cutoff of 3% dissimilarity. The clusters were used to calculate the species-richness estimators and Shannon diversity index. The samples were then randomly normalized to the same number of sequences. For taxonomic assignments, the 16S rRNA gene sequences were classified using the RDP database with a 50% confidence threshold (Cole et al., 2009). For our analysis, we only considered the OTUs that were represented by least three sequences. The sequences from the 48 samples are available at the NCBI Sequence Read Archive under the accession numbers SRR3984611–SRR3984659.

### Data Analysis

For analytical purposes, we previously tested the distribution of the data based on assumptions of normality and homoscedasticity. When the required assumptions for parametric tests were not met, equivalent non-parametric tests were used. To compare the number of copies of the *alkB*, *ndo*, and 16S rRNA genes among the bioremediation treatments, we performed one-way analysis of variance (ANOVA). To compare the number of *alkB*, *ndo*, and 16S rRNA genes between the two sediment layers, we performed Student’s *t*-tests, whereas we performed ANOVA to make comparisons between depths. To evaluate the efficiency of the four bioremediation treatments with respect to total hydrocarbon and alkane degradation, we performed Kruskal-Wallis tests. To evaluate the efficiency of total hydrocarbon and alkane degradation between the two sediment layers, we performed Wilcoxon tests. We conducted these analyses in the R environment (R Core Team, 2015) using the *car* (Fox and Weisberg, 2011), *beeswarm* (Eklund, 2013), and *PMCMR* (Pohlert, 2014) packages. To examine differences among abiotic factors, we performed two-way ANOVA using PAST3 (Hammer et al., 2001). To evaluate samples based on their microbial compositions, we normalized the number of sequences prior to clustering. After that, the singletons and doubletons were removed, which produced samples with slight variations in the number of sequences. Most samples were close to the average of 9406 sequences (the average number of sequences per sample was, respectively, 9300, 9547, 9665, and 9204 for BA, BB, BE, and BT treatments). This shows that there was no strong sequence number bias in a specific treatment, which validates the comparison of the OTU number between the samplesnormalized the number of sequences before clustering. After that, singletons and doubletons were removed, which produced samples with small variations in the number of sequences. Then, we performed a Non-metric Multidimensional Scaling (NMDS) analysis using the Bray-Curtis similarity index, which was calculated as a function of the OTU distribution among the samples using the PC-ORD 6.04 Multivariate Analysis of Ecological Data software (McCune and Mefford, 2011). An outlier sample for the Bioaugmentation + Biostimulation (BB) treatment was retained from the NMDS analysis because it was compressing all of the other samples. Differences in community structure among treatments were tested with a two-way ANOSIM test using PAST3, and a Mantel’s test was performed to test the influence of abiotic factors on community structure with PC-ORD 6.04. To assess whether the treatments and depths had an effect on specific OTUs, we performed blocked indicator species analysis (ISA) (Dufrene and Legendre, 1997). In addition, we performed correlation analysis among the obtained OTUs and abiotic factors. For all tests, we assigned the significance level as α = 0.05.

## Results

### Composition of the Bacterial Consortium

Fifteen anaerobic bacterial strains were isolated in this study, but only two, from the *Desulfovibrio* genus, showed satisfactory growth in the presence of the other consortium strains in antagonistic tests (data not shown). Among the isolated aerobic strains, only four did not show antagonistic activity toward each other. Thus, the hydrocarbon-utilizing bacterial consortium used in the treatments with bioaugmentation conditions was composed of one strain of each of the following genera: *Acinetobacter* (HQ020607), *Azospirillum* (HQ020562), *Paenibacillus* (HQ020564), and *Pseudomonas* (HQ020609), and two isolated strains from the genus *Desulfovibrio* (SAMN06627399).

### Sediment Characterization

Regarding the sediment granulometry, carbon and nitrogen composition/distribution, no differences were observed between treatments or over time (two-way ANOVA, *p* > 0.05), indicating the same physical-chemical conditions across the entire mesocosm. The carbon content was higher in the superficial layer (two-way ANOVA, *F* = 6.07, *p* = 0.01), but the nitrogen content and the C/N ratio did not differ significantly between the two depths. The granulometry of the sediment remained constant throughout the experiment, with no variations observed with respect to treatment or depth.

### Efficiency of Bioremediation Strategies

**FIGURE 2:**
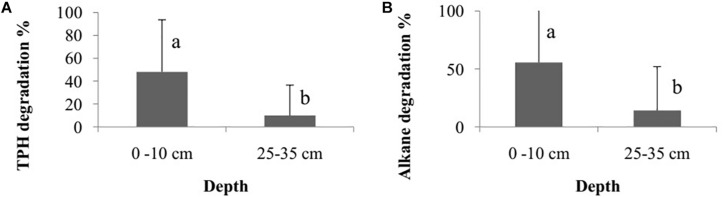
Degradation percentage rate of total petroleum hydrocarbon – TPH **(A)** and alkane **(B)** between different sediment layers (1–10 cm and 25–35 cm). The bars represent the arithmetic mean of all treatments for each depth. The error bars represent the standard deviation. Different letters indicate significant differences between depths.

The degradation percentage rate of total petroleum hydrocarbon (TPH) and alkane did not differ among the bioremediation treatments (Bioattenuation, Bioaugmentation, Biostimulation and Bioaugmentation + Biostimulation) (TPH – Kruskal–Wallis, *p* = 0.35; alkane – Kruskal–Wallis, *p* = 0.30). However, there were differences in degradation rates between the sediment layers (TPH – Wilcoxon, *p* = 0.03, Figure 2A; alkane – Wilcoxon test, *p* < 0.01, Figure 2B) when they were considered as a sum of the degradation percentage of all the treatments. The degradation rates were higher in the superficial sediment (1–10 cm) (TPH – 45.6 ± 41.8%; alkane – 68.1 ± 46.1%) than in the deeper layer (25–35 cm) (TPH – 9.2 ± 29.2%; alkane – 10.0 ± 31.6%) for both TPH and alkane.

### Quantification of Hydrocarbon-Degrading Genes

**FIGURE 3:**
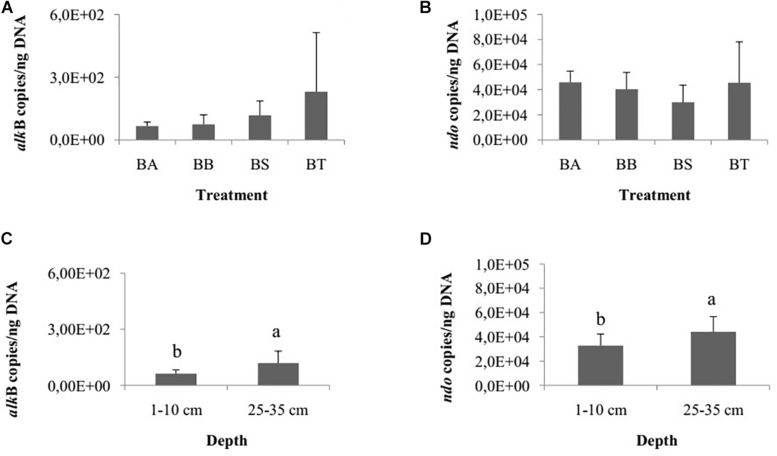
Number of copies of *alk*B **(A)** and *ndo* **(B)** genes/ng DNA among the bioremediation treatments (BA, Bioaugmentation; BB, Biostimulation + Bioaugmentation; BS, Biostimulation; BT, Bioattenuation). The error bars represent the standard deviation. Different letters indicate significant differences between depths. Number of copies of *alk*B **(C)** and *ndo* **(D)** genes between the different sediment layers (1–10 cm and 25–35 cm). The bars represent the arithmetic mean of all treatments for each depth. The error bars represent the standard deviation. Different letters indicate significant differences between depths.

The genes *ass*A and *bss*A, related to anaerobic hydrocarbon degradation, were not detected in our samples. Therefore, we specifically quantified the aerobic genes *alk*B and *ndo*. The quantification of hydrocarbon-degrading genes did not differ among the treatments Bioattenuation, Bioaugmentation, Biostimulation and Bioaugmentation + Biostimulation for *alk*B (one-way ANOVA, *p* = 0.24; Figure 3A) or *ndo* (one-way ANOVA, *p* = 0.14; Figure 3B). However, the number of copies of *alk*B (Student’s *t*-test, *p* = 0.01; Figure 3C) and *ndo* (Student’s *t*-test, *p* = 0.01; Figure 3D) differed between the two sediment layers. The inferior layer had significantly more copies of *alk*B and *ndo* (*alk*B, 1.1E2 ± 6.3E1 copies/ng DNA; *ndo*, 4.4E4 ± 1.2E4 copies/ng DNA) than the superficial layer (*alk*B, 6.2E1 ± 2.0E1 copies/ng DNA; *ndo*, 3.1E4 ± 1.0E4 copies/ng DNA). We did not detect differences for either gene for the different collection times.

### Structure of Bacterial Communities

**FIGURE 4:**
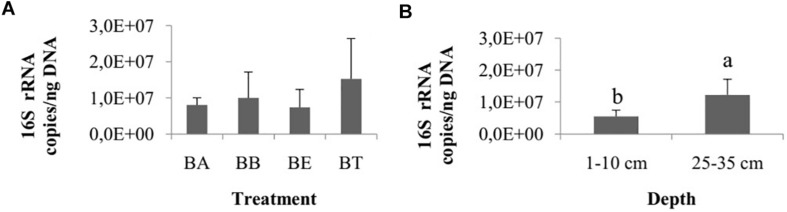
**(A)** Number of copies of 16S rRNA genes among the four bioremediation treatments (BA, Bioaugmentation; BB, Biostimulation + Bioaugmentation; BS, Biostimulation; BT – Bioattenuation.). The error bars represent the standard deviation. **(B)** Number of copies of 16S rRNA genes between different sediment layers. The bars represent the arithmetic mean of all treatments for each depth. The error bars represent the standard deviation. Different letters indicate significant differences.

The number of 16S rRNA gene copies did not differ among treatments (one-way ANOVA, *p* = 0.62; Figure 4A) and collection times (ANOVA, *p* = 0.08), but they did differ between the two sediment layers, with a higher number of copies observed in the 25–35 cm layer (Wilcoxon test, *p* < 0.01; Figure 4B).

**FIGURE 5:**
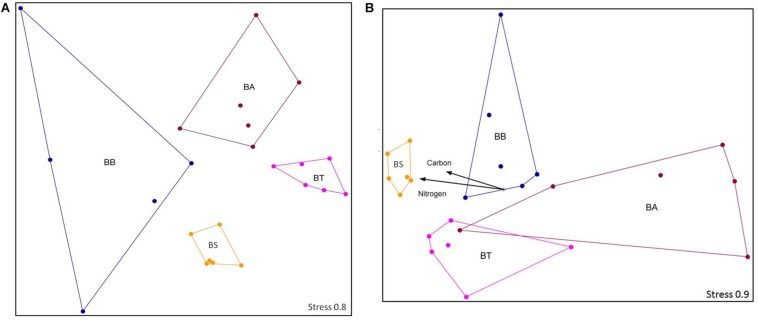
NMS ordination of the sequencing data at the OTU level at depths 1–10 cm **(A)** and 25–35 cm **(B)**. Stress is presented on a scale from 1 to 100. BA, Bioaugmentation; BB, Biostimulation + Bioaugmentation; BS, Biostimulation; BT, Bioattenuation.

From the four treatments, a total of 8000 sequences were obtained after quality trimming and normalization, which clustered into 11,021 OTUs that were classified into 26 phyla, 58 classes, 100 orders, 200 families, and 402 different genera. The OTU structure was significantly influenced by the bioremediation treatments (two-way ANOSIM, *p* < 0.01) and sediment depths (two-way ANOSIM, *p* < 0.01) but was not influenced by collection time (two-way ANOSIM, *p* = 0.50). For the two assayed depths, the OTU number was higher in the 25–35 cm layer (two-way ANOSIM, *p* < 0.01). In this layer, the BS treatment resulted in higher carbon and nitrogen contents (Mantel test, *p* < 0.05), whereas no other abiotic factors stood out for any other treatment (Mantel test, *p* = 0.373) (Figure 5).

**FIGURE 6:**
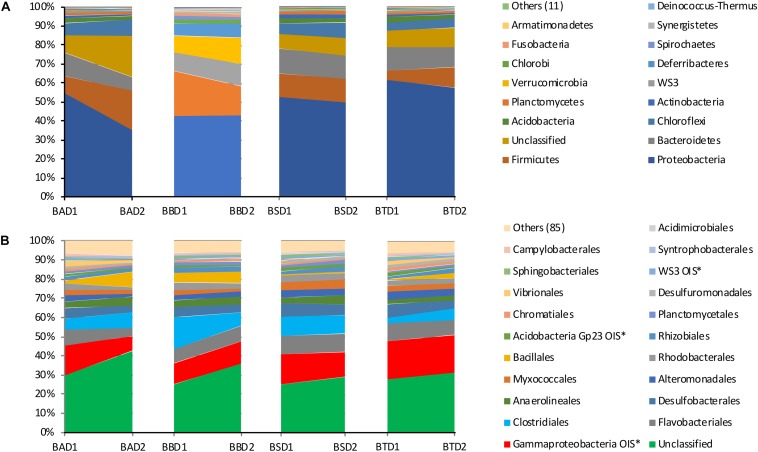
Relative abundance of the bacterial phyla **(A)** and orders **(B)** found in each treatment at each collection depth. BA, bioaugmentation; BB, biostimulation + bioaugmentation; BS, biostimulation; BT, bioattenuation; D1, depth 1–10 cm; D2, depth 25–35 cm.

The community composition at the phylum and order levels did not differ among the treatments, whereas differences were detected between the two sediment layers in all the treatments (Figure 6). The most abundant phyla among all treatments and depths were Proteobacteria, Firmicutes and Bacteroidetes, while the most abundant orders were Gammaproteobacteria OIS^∗^, Flavobacteriales and Clostridiales. A large number of unclassified phyla and orders were also detected. It is worth noting that the most representative fraction of the bacterial community was unclassified at the order level.

The bacterial α-diversity did not vary among significantly samples and depths, as revealed by the observed Shannon H diversity index values and the Chao and Ace richness index values. However, differences in bacterial community composition were revealed by ISA, the results of which showed that 26 different genera were significantly associated with specific bioremediation conditions, 10 of which were related to the BA conditions, nine to the BS conditions and seven to the BT conditions (Table 1). ISA also identified 12 genera that were significantly associated with the two assayed depths (1 to 10 and 15 to 25–35 cm) (Table 1).

**TABLE 1:** Indicator species analysis (ISA) for treatment and depth at the genus level.

**Indicator Species Analysis**				

**Treatment**			**Depth**	
	
**Bioaugmentation**	**Biostimulation**	**Bioattenuation**	**D1 (1–10 cm)**	**D2 (25–35 cm)**
*Bacillus*	*Blastopirellula*	*Formosa*	*Anderseniella*	*Bacillus*
*Caldithrix*	*Desulfosarcina*	*Gp10*	*Clostridium_XI*	*Dehalogenimonas*
*Dehalogenimonas*	*Hoeflea*	*Ignavibacterium*	*Exiguobacterium*	*Desulfobacterium*
*Desulfobacterium*	*Leptolinea*	*Sulfurimonas*	*Formosa*	*Desulfosarcina*
*Gp17*	*Pelagibius*	*Thiohalomonas*	*Gp10*	*Gp17*
*Gp18*	*Rhodopirellula*	*Thiohalophilus*	*Gp23*	*Gp18*
*Gp21*	*Robiginitalea*	*Thioprofundum*	*Ignavibacterium*	*Gp21*
*Lysinibacillus*	*Thalassobius*		*Oceanicola*	*Hoeflea*
*Methylohalomonas*	*Tissierella*		*Sulfurimonas*	*Lysinibacillus*
*Rhodovibrio*			*Thalassobius*	*Methylohalomonas*
			*Thiohalomonas*	*Pelagibius*
			*Thioprofundum*	*Planctomyces*
				*Rhodovibrio*
				*Robiginitalea*
				*Tissierella*

The analysis showed that the distribution of some of the 20 most abundant OTUs correlated with some abiotic factors at D1 and D2. At D1, OTUs from the genus *Desulfopila* (phylum Proteobacteria and class Deltaproteobacteria) correlated positively with nitrogen content (*R* = 0.52, *p* < 0.01) and negatively with alkane (*R* = -0.41, *p* = 0.04). At D2, OTUs from the genera *Actibacter* (phylum Bacteroidetes and class Flavobacteriia) (*R* = 0.68, *p* < 0.01), *Gp23* (phylum Acidobacteria and class Acidobacteria_Gp23) (*R* = 0.64, *p* < 0.01) and *Hailea* (phylum Proteobacteria and class Gammaproteobacteria) (*R* = 0.60, *p* < 0.01) and from the family Desulfobulbaceae (phylum Proteobacteria and class Deltaproteobateria) (*R* = 0.44, *p* = 0.02) and the order Myxococcales (phylum Proteobacteria and class Deltaproteobacteria) (*R* = 0.58, *p* < 0.01) correlated positively with nitrogen content.

## Discussion

### Hydrocarbon Degradation Efficiency of the Bioremediation Strategies

The quantified hydrocarbon rates differed between the two sediment layers, with higher degradation percentages observed at the 1–10 cm depth (D1). This result is probably related to a more anoxic condition on the deeper layers compared with the superficial ones. Generally, hydrocarbon consumption is higher and faster in the presence of oxygen than in its absence (Grishchenkova et al., 2000; Salminen et al., 2004). In a study designed with nitrate-reducing bacteria, the authors identified that, under aerobic conditions (a 10-day experiment), bacteria degraded 20–25% of the hydrocarbon provided, including up to 90–95% of all alkanes analyzed (n-C10–C35). Under anaerobic conditions (a 50-day experiment), the same microorganisms degraded 15–18% of the hydrocarbons, including alkanes (20–25%) and polycyclic aromatic hydrocarbons (15–18%) (Grishchenkova et al., 2000). Other research designed microcosms to study the hydrocarbon consumption by bacterial in an oil contaminated site undergoing a bioattenuation process. It was detected that, in the aerobic microcosms, 31% of the initial oil was removed during a 3-month incubation, while, in the anaerobic ones, on average 44% of the initial oil was removed during a 12-month anaerobic incubation, with removal of some alkanes (n-C11–C15) (Salminen et al., 2004). Despite the difference in depths, the observed oil degradation rates did not differ among the bioremediation treatments. Similar results were also observed in a previous study that used creosote as the contaminant in microcosms, where the degradation rates did not differ among the bioremediation strategies after 6 months of contamination (Simarro et al., 2013). In addition, similar results were observed in a study using crude oil, where the total petroleum hydrocarbon degradation observed using a combined bioaugmentation and biostimulation strategy did not differ from that with bioattenuation after 112 days of contamination (Wu et al., 2017). However, increases in the hydrocarbon degradation rates have also been reported in other sites over short periods of time using bioaugmentation and biostimulation strategies rather than bioattenuation (Yu et al., 2005; Meyer et al., 2014; Müeller et al., 2017). Differences in the obtained results may be explained by studies describing limitations with respect to the biostimulation and bioaugmentation strategies (Boopathy, 2000; Bouchez et al., 2000; Gough and Nielsen, 2016). Regarding biostimulation, the primary challenges are to identify indigenous microbial communities that are capable of degrading target contaminants and to deliver additives in a manner that allows them to be readily available to microorganisms (Boopathy, 2000; Adams et al., 2015). Moreover, at some sites and under some conditions, the addition of nutrients may also promote the growth of microorganisms that can outcompete the native contaminant-degrading organisms (Adams et al., 2015). With respect to the bioaugmentation strategy, the ability of microbes to survive over time depends on multiples variables, such as their attachment to surfaces, substrate availability, predator/prey interactions, competition, and other components that can be associated with the site, environmental conditions and the selected microbial consortia (Bouchez et al., 2000; Gough and Nielsen, 2016). Additionally, it has been suggested that the capacity of soil microbial communities to degrade hydrocarbons depends on their previous exposure to the contaminant (Sánchez et al., 2004; Johnsen and Karlson, 2005). Given that our studied area is a pristine mangrove, with no previous oil contamination reported, our results corroborate the thesis that previous exposure of microorganisms to oil is not needed to stimulate its degradation.

As we detected high levels of hydrocarbon degradation for all treatments and found no differences of degradation rates among them all, our results suggest a high efficiency of the natural bioattenuation for oil consumption in the studied mangrove sediments, revealing the potential of the bioattenuation for environmental decontamination for this site.

Besides this, the presence of the genes *alk*B and *ndo* in all treatments and depths reinforces the idea that native microbial communities have the potential to degrade petroleum hydrocarbons. Our results showed that the number of *alk*B and *ndo* gene copies did not differ among the bioremediation treatments and collection times, although they were detected in higher abundances in the deeper sediment layer. This is a noteworthy finding, given that these genes are associated with aerobic hydrocarbon degradation. As, regarding the evaluated paremeters, the carbon content was the only one that differed between sediment layers, with higher values observed in the superficial layers, we suggest that other environmental conditions, such as dissolved oxygen, pH, temperature and the concentrations of other nutrient may affect the abundances of microorganisms containing these genes in mangrove superficial sediment layers. Furthermore, AlkB-encoding genes are widespread in nature, and although they share considerable sequence homology, the *alk*B gene sequence varies greatly within bacteria harboring this gene (Kuhn et al., 2009; Jurelevicius et al., 2013; Viggor et al., 2015). The same phenomenon has been observed for the *ndo* gene (Larkin et al., 1999; Kasai et al., 2003; Gomes et al., 2007). Thus, the design of broad-ranging *alk*B and *ndo* primers is a challenge. Despite this difficulty and the existence of other mechanisms of aerobic alkane and PHA degradation, degradation routes that involve the *alk*B and *ndo* gene products have been investigated in great detail (Bellinaso et al., 2003; Kloos et al., 2006; Gomes et al., 2007; Flocco et al., 2009; Touvora et al., 2016; Long et al., 2017) and are some of the most commonly studied routes of alkane and PHA degradation in natural environments.

We could not detect the presence of the *ass*A and *bss*A genes in any of our samples. Nevertheless, this does not mean that anaerobic hydrocarbon-degrading microorganisms are not present in the studied area. In fact, hydrocarbon degradation was detected in D2, a sediment layer considered to be anaerobic, which suggest the presence of anaerobic hydrocarbon degraders in the studied mangrove. Our failure to detect the target anaerobic genes may stem from the fact that microorganisms containing these genes are present at such low abundances in this mangrove that they could not be detected by the standard PCR protocol used. Another possible explanation is that anaerobic hydrocarbon degraders may possess variants of ass/bss sequences, lacking homology to our set of primers. Indigenous microbial communities may also preferentially use other alkane and aromatic hydrocarbon degradation routes catalyzed by enzymes that are not encoded by the target genes assayed with the selected primers, such as carboxylation reactions (Andrade et al., 2012).

To gain a better comprehension of the dynamics of hydrocarbon degradation in mangrove sediments, we suggest the use of more than one set of primers for each of the target genes and that other environmental conditions that may affect microbial communities be investigated, which could improve the detection of each gene and expand knowledge of microorganisms with the potential to degrade hydrocarbon compounds.

### Bacterial Community Structure

The 16S rRNA gene copy number did not differ among the treatments and collection times, whereas they did differ between the assayed depths. In contrast, the OTU number and composition were different for the various treatments and sediment stratum layers, with higher richness observed in the deeper layer. Although this is not a common pattern with respect to soil microbiology, where superficial soil layers can generally be up to one-third more diverse than deeper ones (Fierer et al., 2003; Lamontagne et al., 2003; Eilers et al., 2012; Li et al., 2012; Mendes and Tsai, 2014; Raynaud and Nunan, 2014), other studies (Li et al., 2015) have already identified this pattern in soil, with more diverse bacterial phyla associated with sediments of deep soil layers. The data ordination based on the relative abundances of bacterial groups in each treatment showed an effect of the bioremediation strategies on bacterial communities at the OTU level. Additionally, although the carbon and nitrogen contents and the C/N ratio did not differ among the treatments, and the C content was higher in D1, the bacterial communities in layer D2 from the BS treatment were associated with these factors. Chu et al. (2016) tested the dissimilarity between bacterial communities from surface and subsurface sediment layers and observed that the total carbon content and carbon to nitrogen ratio were good predictors of the microbial community distribution. Additionally, as some nutrients can be primarily retained in the soil surface, the introduction of carbon and nitrogen into the deeper sediment layers can stimulate microbial communities and promote changes in their structure, as noticed for bioremediation strategies involving nitrogen addition (Ramsay et al., 2010; Simarro et al., 2013) and across sediment depths (Mueller et al., 2015), as well as for the introduction of carbon into soil environments (Santos et al., 2011; Li et al., 2012; Simarro et al., 2013).

For all the treatments and depths, the most abundant phyla were Proteobacteria, Firmicutes and Bacteroidetes, with Gammaproteobacteria, Flavobacteriales and Clostridiales being the most common orders. Generally, these are very common and most abundant *taxa* in soil environments (Fierer et al., 2009; Eilers et al., 2012; Mueller et al., 2015; Nogueira et al., 2015). Furthermore, these *taxa* harbor several biological families and genera containing hydrocarbon-degrader microorganisms (Jaekel et al., 2013; Berdugo-Clavijo and Gieg, 2014), which reveals the potential of the mangrove bacteria to degrade these contaminants.

ISA revealed strong differences in the observed taxa in response to the different treatments and between the D1 and D2 layers. Some of the detected genera in the D1 layer, such as *Clostridium* and *Exiguobacterium*, are commonly detected in environments with extreme conditions, with additional detected genera including the sulfur-utilizing bacteria *Thioprofundum* and *Sulfurimonas* (Mori et al., 2011; Han and Perner, 2016) and the genera *Formosa*, *Ignavibacterium*, *Oceanicola*, and *Thalassobius*. In the 25–35 cm layer, the detected groups included the spore-forming bacterial genera *Bacillus* and *Lysinibacillus*; *Desulfobacterium*, and *Desulfosarcina*, which are associated with sulfur metabolism; *Dehalogenimonas*, which has the ability to reductively dehalogenate chlorinated alkanes (Moe et al., 2009); the methylotrophic bacterium *Methylohalomonas* (Sorokin et al., 2007); and *Hoeflea*, *Pelagibius*, *Rhodovibrio*, and *Tissierella*. Some of these genera, such as *Bacillus*, *Clostridium*, *Dehalogenimonas*, *Desulfobacterium*, *Desulfosarcina*, and *Tissierella* are also associated with hydrocarbon degradation (Jaekel et al., 2013; Berdugo-Clavijo and Gieg, 2014). This result, in addition to the higher abundance of OUT at D2, reveals the potential of deeper sediments microorganisms to degrade these contaminants. Besides, we detected *taxa* that were positively correlated to the nitrogen content at different depths. The genus *Desulfopila* was detected at D1, while the taxa detected at D2 included the genera *Actibacter*, *Gp23* and *Hailea*; the family Desulfobulbaceae; and order Myxococcales, some members of which also have hydrocarbon degradation functions, such as members of the family Desulfobulbaceae (Laban et al., 2015).

Investigations to better understand the dynamics of bioremediation are crucial in the design of efficient, less costly and environmentally sound cleaning strategies. In this study, we describe the use of *in situ* mesocosms to study the effects of bioremediation strategies for petroleum hydrocarbons to evaluate changes in the microbial community during the process and assess their potential for oil degradation. We believe that closer interactions between bench and field studies would be beneficial by contributing to a better scientific understanding of microbial processes and capabilities in specific areas. Such knowledge can contribute to the development of efficient bioremediation processes at different locations.

## Data Availability

The datasets generated for this study can be accessed from NCBI Sequence Read Archive, SRR3984611–SRR3984659.

## Author Contributions

LM, EM, RP, and AR conceived and designed the experiments. LM, JP, and EM performed the experiments. LM, DL, CR, RP, and AR analyzed the data. JP, RP, and AR contributed to the reagents, materials, and analysis tools. LM, CR, RP, and AR wrote the manuscript.

## Conflict of Interest Statement

JP was employed by company Petrobras. The remaining authors declare that the research was conducted in the absence of any commercial or financial relationships that could be construed as a potential conflict of interest.

## Footnotes

1 http://blast.ncbi.nlm.nih.gov/

2 www.oceanus.bio.br

3 www.mrdnalab.com
