# Supplementary figures and images for: Tracking Mangrove Oil Bioremediation Approaches and Bacterial Diversity at Different Depths in an in situ Mesocosms System
Source: Front Microbiol. 2019 Sep 13;10:2107. doi: 10.3389/fmicb.2019.02107 (PMC6753392; doi:10.3389/fmicb.2019.02107)

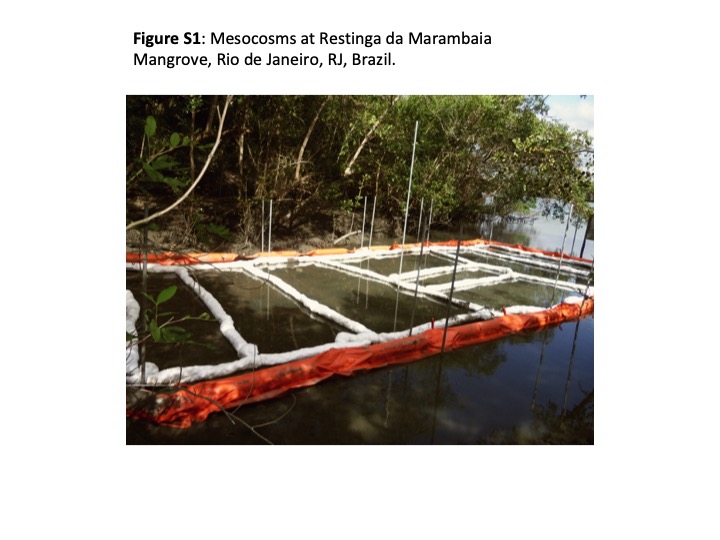

Supplement: Supplementary file 1 [file Image_1.JPEG]

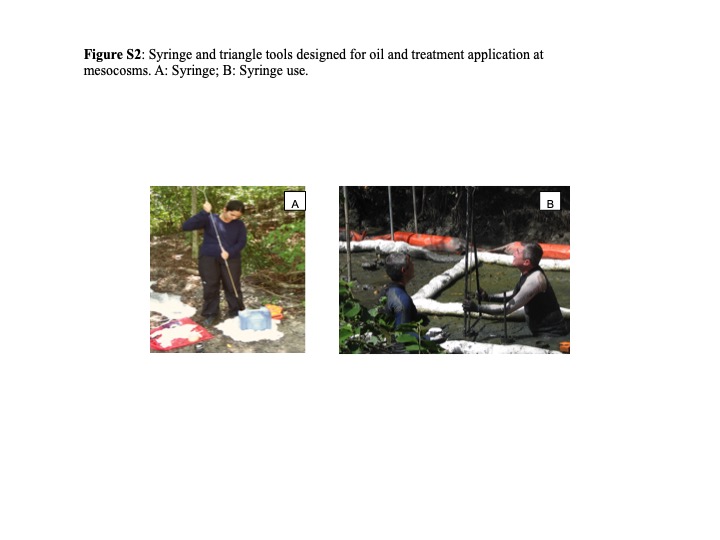

Supplement: Supplementary file 2 [file Image_2.JPEG]
