# Supplementary material for: Tracking Mangrove Oil Bioremediation Approaches and Bacterial Diversity at Different Depths in an in situ Mesocosms System
Source: Front Microbiol. 2019 Sep 13;10:2107. doi: 10.3389/fmicb.2019.02107 (PMC6753392; doi:10.3389/fmicb.2019.02107)
Supplement: Supplementary file 3 [file Table_1.DOCX]

**Supplementary Material**

| **Genes** | **Primers** | **Primers sequences 5’-3’** | **Thermocycling conditions** | **References** |
| --- | --- | --- | --- | --- |
|  |  |  |  |  |
| ***alkB*** | alkB1-f | AAY ACN GCN CAY GAR CTN GGN CAY AA | 94 °C for 5 min, 35 cycles of 94 °C for 1 min, 50 °C for 1 min and | Kloos *et al*., 2006 |
|  | alkB1-r | GCR TGR TGR TCN GAR TGN CGY TG | 72 °C for 1 min, final extension of 72 °C for 3 min |  |
| ***ndo*** | NAPH1F | TGG CTT TTC YTS ACB CAT G | 94 °C for 7 min, 35 cycles of 94 °C for 1 min, 53 °C for 1 min and | Gomes *et al*., 2007 |
|  | NAPH1R | DGR CAT STC TTT TTC BAC | 72 °C for 2 min, final extension of 72 °C for 10 min |  |
|  | NAPH2F | TAT CAC GGC TGG | 94 °C for 5 min, 32 cycles of 95 °C for 1 min, 51 °C for 1,5 min and | Gomes *et al*., 2007 |
|  | NAPH2R | ATS TCT TTT TCB AC | 72 °C for 2 min, final extension of 72 °C for 10 min |  |
| ***assA*** | 7766F | TGT AAC GGC ATG ACC ATT GCG CT | 94 °C for 5 min, 35 cycles of 94 °C for 1 min, 56 °C for 1 min and | Netzer *et al*., 2013 |
|  | 8543R | TC GTC RTT GCC CCA YTT NGG | 72 °C for 1 min, final extension of 72 °C for 5 min |  |
| ***bssA*** | 7772F | GAC ATG ACC GAC GCS ATY CT | 94 °C for 3 min, 35 cycles of 94 °C for 30 s, 52 °C for 30 s and 72 °C | Netzer *et al*., 2013 |
|  | 8546R | TC GTC GTC RTT GCC CCA YTT | for 1 min, final extension of 72 °C for 5 min |  |
| **16S rRNA** | 357F | CTA CGG GRS GCA G | 95 °C for 3 min, 35 cycles of 95 °C for 30 s, 55 °C for 1 min and 72 | Muyzer et al., 1993 |
|  | 529R | CGC GGC TGC TGG CAG | °C for 1 min, final extension of 72 °C for 10 min |  |
|  |  |  |  |  |

Table S1. Primers and PCR conditions for the hydrocarbon-degrading and 16S rRNA genes.
